# A New Form of Train Detection as a Solution to Improve Level Crossing Closing Time

**DOI:** 10.3390/s23146619

**Published:** 2023-07-23

**Authors:** Michał Zawodny, Maciej Kruszyna, Wojciech Kazimierz Szczepanek, Mariusz Korzeń

**Affiliations:** Faculty of Civil Engineering, Wrocław University of Science and Technology (Politechnika Wrocławska), 50-370 Wrocław, Poland; maciej.kruszyna@pwr.edu.pl (M.K.); wojciech.szczepanek@pwr.edu.pl (W.K.S.); mariusz.korzen@pwr.edu.pl (M.K.)

**Keywords:** V2I, IoT, grade crossing, level crossing, railway, detection, ITS, radar, LIDAR, simulation

## Abstract

The critical points on the rail and road network are their intersections, i.e., level crossings. During a train crossing, car traffic is stopped. This reduces the fluidity of traffic on the road and, consequently, can cause congestion. The problem increases with the number of cars and trains. Frequently, due to national regulations, level crossing closure times are long. It is mainly dictated by safety issues. Building two-level intersections is not always a good solution, mainly because of the high cost of implementation. In the article, the authors proposed the use of sensors to reduce level crossing closure times and improve the Level of Service on the road network. The analyzed railroad lines are local agglomeration lines, mainly due to safety (low speed of commuter trains) and high impact on the road network. The sensors proposed in the article are based on radar/LIDAR. Formulas similar to HCM methods are proposed, which can be implemented in a railroad crossing controller. Simulations using the PTV Vissim program are carried out and the results are worked out based on the obtained data. The considered method can reduce the level crossing closure time by 68.6%, thereby increasing the Level of Service on roads near railroads.

## 1. Introduction

Currently, level crossings have a large margin of safety; therefore, they cause losses in the level of service. Longer delays can lead to dangerous situations when impatient drivers may break traffic rules. In this paper, the authors propose methods to reduce the time of crossing closure. Due to safety concerns, the proposed changes are first considered for low-speed suburban rail lines.

The proposition is to change a way of looking at level crossings similar to that of a tram line with a priority on intersections with ITS systems. Achievement of the goal can be obtained by the same measure—detection of the train. Nowadays, trains are detected in a particular place, and a crossing is closed at the moment of detection. The limitation of the existing method is detecting only the fixed appearance point of the train at the track section near the level crossing, without any movement characteristics of the train. Recently used track circuits have a few points of failure, which are impossible to detect immediately. The track circuit malfunctions when the rail is broken, and cables are at ground level; therefore, the system is prone to theft. Moreover, in case of ice, contaminated ballast, leaves or flooding can malfunction the circuit. The proposed method detects not only the train but also the speed and acceleration. Although trams have similar speeds at intersections, the speed of the train can be different depending on its type (suburban, regional, intercity) due to the type of vehicle and the number of stops. In addition, the rail network is used by cargo trains, which usually operate at much slower speeds than passenger trains. A level crossing with a detection point adjusted to the highest possible train speed is inefficient, especially when a stop is behind a detection point, which causes the level crossing to close when the train is standing next to the platform.

Recent proposed safety measures in level crossings consider the use of strobe lights on train locomotives [1]. Many studies have been conducted on obstacle detection at level crossings; for example, in [2], using surveillance cameras already mounted on level crossings to detect road vehicles in the collision area. In [3], the authors suggest using CCTV cameras and radars directed into the crossing area as a secondary safety tool for detecting pedestrians. The CCTV cameras can be used in traffic flow analysis [4]. The deep learning tools for detecting obstacles can be used by the locomotive as well [5], and they can also detect obstacles near tracks [6] and during shunting works [7]. Deep learning can also be used to study traffic delays [8]. A review of deep learning methods in rail is conducted in the paper [9].

Another type of safety measure is to warn drivers about a passive level crossing via V2I (Vehicle-to-Infrastructure) messages [10]. The effectiveness of this method requires a high percentage of vehicles equipped with V2X (Vehicle-to-Everything) devices. Such a scenario with autonomous vehicles is proposed in [11]. Communication with the vehicles must be carried out in a protected network such as the Global System for Mobile Communications–Railway (GSM-R) [12], in WAVE standard architecture [13]. However, the GSM-R standard has severe limitations [14]. Recent studies have proposed more advanced solutions [15,16,17]. The Internet of Railways network is proposed in [18].

The classic ways to detect a train are a track circuit or an axle counter [19]. Detection measures can be upgraded with the use of Fiber Bragg Gratings [20]. In the paper, we suggest using radars to detect trains; the conclusions of studies in [21] indicate that the radar solution produces better results than vibration or voice-based sensors. Based on the [21] conclusions, we also proposed LIDAR as another promising solution. The technology is not mentioned in the research; however, we proposed the LIDAR as a sensor without limitations of less effective researched tools. A similar study on reducing the time to close a level crossing, but without the use of radar, is proposed in [22].

Geospatial modelling methods proved that the behavior of drivers violating traffic rules at level crossings is different in parts of the USA [23]. In a study [24], the use of phones by drivers in the vicinity of a level crossing was measured. Drivers who are able to see the train in the distance are more likely to trespass into a closed level crossing [25]. GIS-based methods can be used as well for identification of most dangerous level crossings to implement safety measures [26]. Probabilistic methods are proposed in [27]. The population of the area near level crossings is added into probability methods in [28]. The meta-analysis about the safety of level crossings was carried out in [29]. One of the conclusions is that there is little progress in systems thinking approaches to understand risks at level crossings.

The Road Safety Thematic Report [30] concluded that, in the EU, fatalities resulting from level crossings represent only 1% of all road-related fatalities. In Poland, 44.9% of level crossings have no signals or barriers (category D) [31]. However, these level crossings account for 65.7% of crashes. Therefore, the safety of other level crossings is higher, so a reduction in closure times hypothetically can be reduced without severe safety concerns. Reducing the time of the level crossing closure can increase safety [32] and lower social costs such as air pollution [33]. The study [34] conducted in India proved that the closure time of the level crossing has a significant impact on road-user Level-of-Service perception. A case study about traffic delays and ways of reducing them was carried out in [35]. The problem of reduced Level of Service could be solved by level separation with a bridge or tunnel. However, this solution is expensive [36,37]. For example, the removal of 75 level crossings in Melbourne will cost at least A$14.8 billion [38]. Moreover, due to geometric problems, the building of an intersection with two levels is not possible at every location. Even on two-leveled intersections with rail, dangerous situations can happen [39]. Level separation can provide additional unexpected results such as suicide prevention [40]. Due to the costs of building two-leveled intersections, a method for selecting the most important ones for rebuild is proposed in [41]. The other studies concerning level crossing safety calculated safe visibility triangles measure methods [42,43]. Safe distances for train detection are also proposed in [44]. The paper acknowledges even cargo trains, and regional urban lanes can also be used for slower freight trains [45].

A similar study with simulation was conducted in [46]. The study proposed warning time-saving methods, such as restricting large vehicles, preemption on near-road light signals, reducing minimum warning times, and road opening times. The study briefly suggested methods similar to that proposed in the paper, yet without technical aspects, which are further considered in our paper with case study. The authors in [47] proposed optimalization of level crossing algorithms, without using new tools for detecting trains. The study indicates that improvements of existing systems are possible even when the level crossing is based on track circuits. Both studies show the importance of level crossing time reductions with a variety of places with the possibility of changes in order to reduce closure time. Our study develops the ideas further, with proposals of using specific technology that can provide even better results.

## 2. Materials and Methods

As sensors used for detecting trains, we suggest using radar or LIDAR. The change is needed to detect the speed and acceleration of the incoming train. Details about radar in rail application are mentioned in the literature review. The radar application proposed in this paper takes into account the detection of the actual speed and acceleration of the train based on successive measurements. Therefore, the radar must be advanced enough to measure the speed of the train in high frequency, not only the presence of the train. In the paper, we do not propose an exact hardware piece, as it should be considered based on local conditions relating to availability and the producer, and to the geometrics of the rail line. The detection point should not be closer than the point of the time-saving closure of the level crossing for a train at the maximum allowed speed on the line. Accordingly, a detection point can be set at a place of a recent detection mechanism of the train if it is based on the train’s maximum speed. Depending on the track geometry and radar/LIDAR detection range, the radar/LIDAR can be placed even on the level crossing. The motivation for using such methods stems from problems with aging rolling stock and various rail line users. We suppose that the aging rolling stock will not be equipped with IoT devices, or the devices will be limited to only measure technical conditions, as the purpose of using old rolling stock is the need for investment in a new one. The radar/LIDAR solution does not require investments in existing rolling stock. The proposed technology will detect trains regardless of the age. The only changes made would be to the rail infrastructure, owned by one company; therefore, it is possible to implement the method at any time. Moreover, the technology can be used only in crucial level crossings, which are bottlenecks in the transport systems. Old track-circuit-based level crossings could possibly stay in locations with high road capacity. The solution would be the most cost-effective and therefore could improve capacity, even with limited resources.

A more advanced solution would be the application of IoT systems. This is similar to the V2X proposal for autonomous vehicles, which allows them to cross intersections. Via V2X, a train equipped with sensors will transmit information about its speed, acceleration and brakes technical condition. The condition is needed in case of a rail stop in the vicinity of a level crossing, to remove the safe closing margin in case of brake failure. The authors recommend using the GSM-R network or a newer one to obtain low latency of the signal. The range of the GSM network is approximately 10 km at maximum, in case the range needed is lower than 1 km. Preferable solutions would be LTE-R, 5G, or IoR to lower latency of the signal. The signal should be based on the WAVE IEEE standard in terms of architecture and operation. This architecture is needed to provide safe and uninterrupted communication. The motivation of an advanced IoT solution is an increase in IoT solutions in transportation. The IoT devices will be necessary to allow automated vehicle traffic; therefore, with the percentage increase of automated vehicles, it will be possible to connect vehicles directly with the level crossing, without the need for detecting closed crossing signals. This solution will be most suitable for future forms of the traffic. Even without the automated vehicles, the V2X (Vehicle-to-Everything) solutions for human-operated vehicles are in the development and applications stage. In future, it will be possible to connect the vehicles and the trains directly via V2V (Vehicle-to-Vehicle) communication, without a need to connect with the level crossing beforehand. From the technical point of view, IoT solutions can provide real-time information about the train movement to the level crossing, hence the precision of closing the crossing.

**Figure 1:**
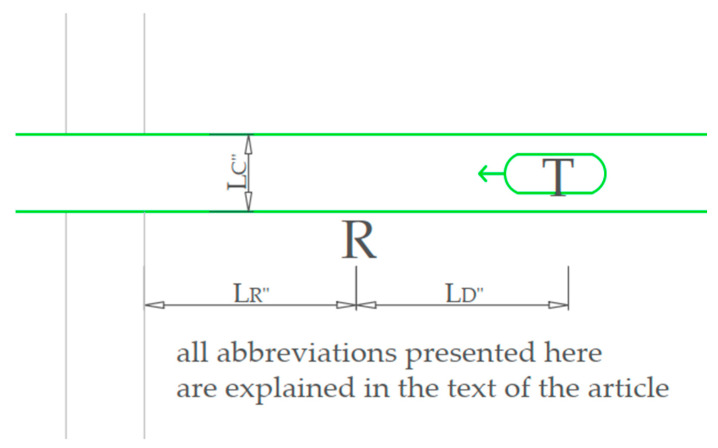
This figure shows the lengths used in calculations.

In summary, the IoT solution has more technical advantages, and it is more promising over the long time horizon. However, we proposed and further describe the first methodology as a solution possible to implement in the current state of transportation, and the solution based on radar/LIDAR has more points of uncertainty than the real-time information exchange possible with IoT devices. Therefore, it was necessary to describe solutions to all points of uncertainty in the paper. Also the main motivation of proposing less advanced solutions is the possibility of installing it now, without any additional investments that will take time to implement before method application in real infrastructure, as it is a side upgrade of an existing system. According to the literature review, the radar or LIDAR are the most promising types of hardware used for detecting trains; therefore, we decided to propose them in the paper. Crucial lengths used in the following calculations are shown in Figure 1.

Key elements:
R—radar/LIDAR location.
T—train.
L_R_—length of radar location—between the beginning of the crossing (first possible collision point with vehicle) and the radar, when the radar is placed at the crossing, can be equal to zero.
L_D_—length of detection—between the radar and the first detection point of the train.
L_C_—length of crossing—between the first and the last point of collision of the road vehicle and the load gauge of the railway line.
L_S_—length of signals—between the first points of the train loading gauge and the location of level crossing signaling lights from two sides of the track.
L_V_—length of the longest allowed road vehicle.
V_V_—speed limit for vehicles.
V_T_—detected train speed.
V_Max_—maximum speed limit for trains.
A_T_—detected acceleration of the train (cannot be negative even when the train is decelerating).
T_A_—arrival time—time from the train first detection to collision with the road gauge.
T_E_—evacuation time—time needed for the last road vehicle to leave the crossing.
T_C_—closing time—time of the crossing closure after detecting the train.

The formulas used in the paper are simplified for need of implementation in infrastructure. The method is possible to implement by infrastructure administrators. All needed data are available to obtain in the design or construction phase of investment. Moreover, the calculations are similar to classical intersection traffic light calculations. Authors suggests a change in thinking about closing level crossings to those similar with priorities for trams in intersections with ITS systems, however with wider safety margins.

(1) $$T_{A}=\frac{L_{R}+L_{D}}{V_{T}}+\sqrt{\frac{2\cdot (L_{R}+L_{D})}{A_{T}}}$$

with limitations:

(2) $$V_{T}\leq \frac{L_{R}+L_{D}}{V_{\mathrm{Max}}}$$

(3) $$A_{T}\geq 0$$

Equation (2) does not allow to lengthen the closure time for longer than it is possible on the line. Equation (3) is applied only when the radar is not directly on the level crossing to avoid situations in which the train will stop decelerating behind the radar range, which would reduce the closure time to a dangerous level.

(4) $$T_{E}=\frac{L_{C}+L_{S}+L_{V}}{V_{V}}$$

(5) $$T_{C}=T_{A}-T_{E}$$

A lower closure time can raise safety concerns. Therefore, authors suggest using CCTV cameras for detecting road vehicles in the collision zone. The use of CCTV is not within the scope of the paper. Research studies about the system are in the literature review. Information about the obstacle can be transferred via V2I systems based on GSM-R or newer. Additionally, to increase safety and the possibility of stopping the train, authors will implement the concept on regional low-speed lines near agglomerations. After further implementation study, the concept can be implemented on high-speed rail lines.

Another significant element needed for improving the closing time is the detection of the train as soon as it leaves the gauge of the road on the level crossing. To detect the exact time of the train leaving, the abovementioned CCTV can be utilized. As soon as the train leaves the level crossing, the gates will be risen, and after, the gates light signaling will stop. Based on a review of the literature and the measurements conducted, this time is approximately 6 s.

In the case of using IoT solutions instead of radar/LIDAR, object detection via CCTV cameras at a level crossing can be used for emergency stop of the train in the case of a vehicle in the level crossing. Even when a full stop of the train is not possible, reduction of speed can lead to lower consequences of crash.

## 3. Simulation

**Figure 2:**
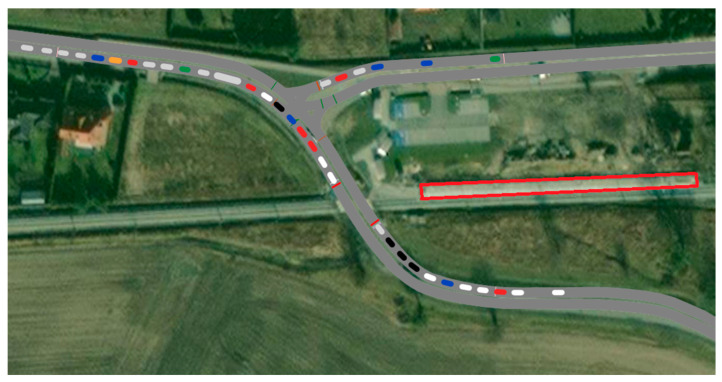
This figure shows the situation of the road network within the level crossing at the time of its closure. The area of the railway stop is marked in a red rectangle.

PTV Vissim is traffic simulation software that digitally reproduces the traffic patterns of all road users. Using this program, you can check the parameters of traffic conditions. In the analyzed case, it is not the most accurate, but currently there is no software on the market for analyzing road traffic conditions of level railway crossings. The analyses are done on a microscopic level, focused on exact parts of infrastructure. The software is capable of analyzing traffic behavior with a variety of customizable parameters. Moreover, it can analyze signal control; these two aspects are crucial in analyzing the case study. To verify the proposal, we carried out several simulations using PTV Vissim 2021 (SP 13) software. The first case is the current state, which is used to make comparisons. Second, the simulation type is a proposed state with grade crossing closure reduction. The analyzed level crossing is located in southwestern Poland in the village of Nadolice Wielkie. The village is part of the Wroclaw agglomeration, with a population of about 1 million. Another study on the agglomeration and the railway line was conducted in [48] using innovative heuristic tool NOAH for public transport changes analysis. The analyzed railroad line carries suburban and cargo traffic. The area is selected based on where the railway stop has an impact on the closed part of the level crossing. The situation is shown in Figure 2.

The following parameters are measured on the railway level crossing, which are necessary to perform the simulation:

1. The rail level crossing closure time;
2. Traffic volume road traffic;
3. Time of arrival of the train at the level crossing.

Table 1 and Table 2 present the numerical values from field measurement, which are used in simulations.

The traffic value intensities in Table 1 are the peak traffic at the time of the measurement divided into quarters of an hour. Measurements are taken on Wednesday from 1:00 p.m. to 4:00 p.m. During this time, 3 passages of trains stopping at the stop in front of the railway level crossing are measured and presented in Table 2. When creating the simulation in the PTV Vissim software, the most unfavorable case is assumed at the closure of the railway level crossing. First, measurement of the railway level crossing closure time from Table 2 is assumed, while the assumed values of road traffic intensity are presented in Table 1, as “In total”. Based on the arrival time of the train through the crossing from the activation of the red signal:

(6) $$T_{E}=\frac{3.5\;m+2\times 4\;m+12\;m}{30\;\mathrm{km}/h}=2.82\;s$$

The assumptions are made in the case of a train stopping at a stop that is 50 m away from the crossing. A distance of 50 m is too short to close the level crossing when starting the train. To calculate, we assumed that passenger exchange is not less than 20 s based on civil engineering design assumptions. When the detector notices a stop of the train, the gates at the level crossing will close. The train acceleration is assumed to be 1.0 m/s^2^ and the length 120 m. One track gauge is assumed as 3.5 m, a distance from the barriers as 4 m on each side. Therefore, L_c_ is 11.5 m. With L_v_ as 12 m, T_E_ is 2.82 s with the vehicle speed of 30 km/h; the value is lowered due to safety reasons.

However, the considered case is near a rail stop in the village; therefore, as T_E_ we calculated the speed of pedestrians. The assumed pedestrian speed is 1.4 m/s, and the length of the pedestrian is calculated as 0 s as in the Highway Capacity Manual. By using Equation (4), the escape time will be 8.21 s, rounded up to 9 s. The assumed time of closing the gates is 6 s, based on measurements. In addition, we add 3 s as the time when the driver or pedestrian will not notice or stop in time after the red signal. The safe closing time is 12 s after the red signal (rounded up) plus 6 s for the gate close, that is, 18 s in total. The number is lower than the passenger exchange; therefore, in the case of a train not stopping at the rail stop, the closure time will be lower.

By using an equation similar to (1), the train arrival time is calculated. Furthermore, the length of the train and the length of the road (rounded up to 10 m) are added as follows:

(7) $$T_{\mathrm{Arrival}}=0+\sqrt{\frac{2\cdot (50\;m+120\;m+10\;m)}{1.0\;m/s^{2}}}=18.97\;s\approx 19\;s$$

Right after the train leaves the crossing, the gates will start to open, which will take an additional 6 s. With a 20 s passenger exchange time of closing, a safe closing time of 9 s, plus 19 s for the train to leave the crossing, plus 6 s for opening gates, the reduced time of the closure will take 54 s in the case study. If the sensor does not detect deceleration of the train at the distance needed to stop, the level crossing will close, as in the case without a rail stop. In the case study, this is similar to a train incoming from the west side of rail line. However, if the railway stop is closer to the level crossing, and the speed of the train is high enough for the need to close the crossing before the moment of detecting deceleration, we suggest using only light signaling at the crossing. Traffic stops will be minimal, and it will be possible to pass vehicles after detection of deceleration. When the sensor notices no deceleration before the railway stop, the gates will be lowered to additional safety.

## 4. Results

Simulations of two variants are made based on the PTV Vissim software, focused on determining the length of the maximum vehicle queue and average delay. Additionally, percentage changes are presented comparing the existing variants and the predicted variant. The results obtained for each case are summarized in Table 3, Table 4 and Table 5.

Due to the reduction of the time of complete closure of the passage through the use of remote detection, a significant improvement in road traffic conditions has been obtained. The longest time railway level crossing is reduced from 172 s to 54 s. The new variant performs much better than the existing one due to the time reduction by 118 s. The value decreased by 68.6%, while maintaining traffic safety during closure of the railway level crossing. The results simulations that show this are as follows. At the north–west intel, the maximum vehicle queue is reduced by 71.1% and average delay is reduced by 87.8%. In the south–east intel, these values are 62.7% and 78.3%, respectively. The most improved traffic conditions at the last north–east intel and values are reduced by 82.2% and 90.1%, respectively. The greatest improvement occurs at the last intel, because after opening the level crossing, other intel vehicles block the relations.

Moreover, traffic intensity is the lowest on the level 244 [V/h] in the case. The second important intel is North–West, which, in the similar situation, is blocked by the opposite relation. However, traffic intensity is greater by 61.3% compared to the previous case. The last intel does not have collision relations, so the only factor improved on the case is reduced total time closure of the railway level crossing. Summarizing the above part, the maximum vehicle queue has decreased on the level between 62.7% and 82.2% and average delay reduced on the level between 78.3% and 90.1%. This is a significant improvement in traffic conditions, which is at a minimum level of 60%. Taking into account the results presented in Table 4 and Table 5, it can be seen that the improvement for the north–west intel is comparable to the described case. This is due to the highest traffic volume and significant left turn dependence. The similar problem exists at intel north–east. The most important change can be seen at intel south–east as the relation has no collisions. In the existing state between the worst case and ideal case differences, values average delay is 14.7% and maximum vehicle queue is 7.8%. This does not change the fact that these are still small differences compared to the suggested solution.

## 5. Conclusions

The article proposes a new way of detecting trains at level crossings. The new detection form will allow to reduce the time of crossing closure by calculations based on speed and acceleration of the train. Recent methods of detecting trains do not measure their speed, only the detection in a fixed point in the railway. The detection method proposed in the article is based on radar/LIDAR in case of no interference in the system inside trains. A more advanced solution consists of IoT-based IoR measures inside the train with suggested GSM-R or newer connection. An advanced solution is similar to V2X systems considered in autonomous vehicle traffic management. In the paper are proposed formulas similar to HCM methods that can be implemented in the controller of the level crossing. Based on conducted traffic measures near level crossings in the village within the city agglomeration, we concluded simulations in the software PTV Vissim. The authors measured real closing time and compared it to a scenario with proposed changes. The worst-case closing time is reduced to 54 s, from the measured 172 s, reducing closing time by 68.6%. Based on the case, the proposed method allowed to reduce time delays of road vehicles by not less than 75% and reduced the maximum vehicle queue by not less than 60%. A similar study [46] had a reduction in times by 7% up to 57%; therefore, it shows that the proposed changes of train detection methods in the paper can reduce the traffic delays by greater value. Better results were possible due to proposed methodology, consisting of using new sensors to detect characteristics of the train movement.

The authors propose that the changes begin at the agglomeration line due to safety reasons. Usually agglomeration rail lines use lighter rolling stock with lower speeds. The real-world application needs to consult with the rail line administrator. Depending on the law within the level crossing area, with possible law changes allowing reduction of the safety margins, the radar/LIDAR needs only changes in the infrastructure, by replacing the old train detection system, and modification of the level crossing controller. IoT solution requires investments in rolling stock. The hardware application in the real world will be removing track circuits in the level crossing area; instead, the radar or LIDAR will be mounted, for safety reasons at few meters height (we suggest at least 3 m, to lower the possibility of theft or vandalism acts). The hardware connection with level crossing controller can be made through classical cables, or optical fibers. We suggest placing them inside pole used for radar/LIDAR and further buried into the ballast to provide further safety from theft. Changes of the controller will be minimal, only into the algorithm of the crossing, by adding formulas described in the paper. Application of IoT-based—advanced solution will make detection hardware unnecessary, it can be removed. However it will be necessary to equip all rolling stock with sensors detecting and reporting to the level crossing controller. The controller of the level crossing must be equipped with antennas able to receive signals from the sensors in the train in a safe distance; in the paper, we suggested possible technologies of communication. The real-world application can be a mixed solution. During equipping the rolling stock with IoT devices, the old train detection hardware, proposed radar/LIDAR or even track circuits, can detect the trains not equipped with IoT, and the level crossing controller will ignore measurements from the hardware when the train equipped with IoT will be detected. The proposed method is open to changes, as it is an introduction to discussion about the need for new level crossing systems, and reduction of high closure times due to old approaches to detecting trains at the crossings. According to literature review, lower closure time can lead to lower violation of traffic rules at level crossings. Our proposition mainly has three changes in the existing technology. Firstly we change train detection hardware from detecting the train at a point to detecting speed and acceleration. In the literature review, we have found that the radar is the most promising sensor, as novelty we also proposed LIDAR. Secondly, we propose minor changes in the way of closing gates to an absolute minimum with maintaining traffic safety. Thirdly, due to previously mentioned changes, we propose novelistic changes in level crossing controller algorithms and a way to calculate the time of the level crossing closure, we provided detailed calculations, ready to implement in the infrastructure. The calculations are based on existing road traffic signals time calculation, and we made changes necessary for rail implementations. We also implemented simulation based on real measurements; therefore, it was possible to prove efficiency of existing statements on which our research was conducted, as well as the author’s new propositions. Future research will consist of autonomous vehicles scenarios and case study applications of the method in infrastructure.

## Figures and Tables

**Table 1:** Collated traffic intensity in number of vehicles measurements at each intel.

Measurement Time	North–West [Veh]	South–East [Veh]	North–East [Veh]
15 min	96	75	52
15 min	114	100	71
15 min	102	91	64
15 min	86	82	57
In total	398	348	244

**Table 2:** Collated measurements of the railway level crossing time parameters.

Measurement	Total Level Crossing Closure Time [s]	Time of Arrival of the Train from the Activation of the Red Signal [s]
First	172	144
Second	163	125
Third	143	115

**Table 3:** Simulations of existing traffic conditions in first variation and after reduced time closure of the railway level crossing.

Intels	Q_1_ [m]	Q_r_ [m]	Relatively Absolution Q_1r_ [m]	Change Absolution Q_1r_ [%]	d_1_ [s/Veh]	d_r_ [s/Veh]	Relatively Absolution d_1r_ [s/Veh]	Change Absolution d_1r_ [%]
North–West	212.7	61.4	151.3	71.1	44.2	5.4	38.8	87.8
South–East	113.9	42.5	71.4	62.7	12.9	2.8	10.1	78.3
North–East	60.2	10.7	49.5	82.2	20.2	2.0	18.2	90.1

Q_1_—Maximum vehicle queue in existing conditions, where level crossing closure time is 172 s in existing simulation. Q_r_—Maximum vehicle queue after reduced time closure simulation, where level crossing closure time is 54 s after reduced time. d_1_—Average delay in existing conditions. d_r_—Average delay after reduced time closure simulation.

**Table 4:** Simulations of existing traffic conditions in second variation and after reduced time closure of the railway level crossing.

Intels	Q_2_ [m]	Q_r_ [m]	Relatively Absolution Q_2r_ [m]	Change Absolution Q_2r_ [%]	d_2_ [s/Veh]	d_r_ [s/Veh]	Relatively Absolution d_2r_ [s/Veh]	Change Absolution d_2r_ [%]
North–West	212.1	61.4	150.7	71.1	41.6	5.4	36.2	87.0
South–East	107.0	42.5	64.5	60.3	11.1	2.8	8.3	74.8
North–East	59.5	10.7	48.8	82.0	15.5	2.0	13.5	87.1

Q_2_—Maximum vehicle queue in existing conditions, where level crossing closure time is 163 s in existing simulation. Q_r_—Maximum vehicle queue after reduced time closure simulation, where level crossing closure time is 54 s after reduced time. d_2_—Average delay in existing conditions. d_r_—Average delay after reduced time closure simulation.

**Table 5:** Simulations of existing traffic conditions in third variation and after reduced time closure of the railway level crossing.

Intels	Q_3_ [m]	Q_r_ [m]	Relatively Absolution Q_1r_ [m]	Change Absolution Q_3r_ [%]	d_3_ [s/Veh]	d_r_ [s/Veh]	Relatively Absolution d_3r_ [s/Veh]	Change Absolution d_3r_ [%]
North–West	208.5	61.4	147.1	70.6	36.9	5.4	31.5	85.4
South–East	94.3	42.5	51.8	54.9	7.7	2.8	4.9	63.6
North–East	56.1	10.7	45.4	80.9	15.1	2.0	13.1	86.8

Q_3_—Maximum vehicle queue in existing conditions, where level crossing closure time is 143 s in existing simulation. Q_r_—Maximum vehicle queue after reduced time closure simulation, where level crossing closure time is 54 s after reduced time. d_3_—Average delay in existing conditions. d_r_—Average delay after reduced time closure simulation.

## Data Availability

Not applicable.
